# Multimodal Monitoring in Large Hemispheric Infarction: Quantitative Electroencephalography Combined With Transcranial Doppler for Prognosis Prediction

**DOI:** 10.3389/fneur.2021.724571

**Published:** 2021-12-08

**Authors:** Yajie Qi, Yingqi Xing, Lijuan Wang, Jie Zhang, Yanting Cao, Li Liu, Ying Chen

**Affiliations:** ^1^Department of Neurology, The First Hospital of Jilin University, Changchun, China; ^2^Department of Neurosurgery, Northern Jiangsu People's Hospital, Clinical Medical College of Yangzhou University, Yangzhou, China; ^3^Department of Vascular Ultrasonography, Xuanwu Hospital, Capital Medical University, Beijing, China; ^4^Beijing Diagnostic Center of Vascular Ultrasound, Beijing, China; ^5^Department of Neurology, Linyi People's Hospital, Linyi, China; ^6^Department of Neurology, Changchun People's Hospital, Changchun, China

**Keywords:** glasgow coma scale, large hemispheric infarction, multimodal monitoring, prognosis, quantitative electroencephalography, transcranial doppler

## Abstract

**Background:** We aimed to explore whether transcranial Doppler (TCD) combined with quantitative electroencephalography (QEEG) can improve prognosis evaluation in patients with a large hemispheric infarction (LHI) and to establish an accurate prognosis prediction model.

**Methods:** We prospectively assessed 90-day mortality in patients with LHI. Brain function was monitored using TCD-QEEG at the bedside of the patient.

**Results:** Of the 59 (55.3 ± 10.6 years; 17 men) enrolled patients, 37 (67.3%) patients died within 90 days. The Cox regression analyses revealed that the Glasgow Coma Scale (GCS) score ≤ 8 [hazard ratio (HR), 3.228; 95% CI, 1.335–7.801; *p* = 0.009], TCD-terminal internal carotid artery as the offending vessel (HR, 3.830; 95% CI, 1.301–11.271; *p* = 0.015), and QEEG-a (delta + theta)/(alpha + beta) ratio ≥ 3 (HR, 3.647; 95% CI, 1.170–11.373; *p* = 0.026) independently predicted survival duration. Combining these three factors yielded an area under the receiver operating characteristic curve of 0.905 and had better predictive accuracy than those of individual variables (*p* < 0.05).

**Conclusion:** TCD and QEEG complement the GCS score to create a reliable multimodal method for monitoring prognosis in patients with LHI.

## Introduction

Large hemispheric infarction (LHI) is a devastating disease associated with high mortality and poor outcomes (1). Severe neurological deficit, disturbance of consciousness, and even cerebral hernia often occur in the early stage of the disease and the mortality rate is as high as 80% in patients receiving conservative treatment (2, 3). Imaging examination can help to identify patients with LHI and facilitate monitoring disease progression (4). However, the complexity and uncertainty of condition of the patients do not allow frequent reexamination and serial imaging might be difficult to perform in critically ill patients (5, 6). Establishing methods to identify the brain functional status of patients with LHI at the bedside, in a non-invasive and objective way in the early stage of disease, and identifying the factors predicting poor prognosis in patients with LHI are urgent clinical goals.

Large hemispheric infarction is usually caused by acute occlusion of the internal carotid artery (ICA) or middle cerebral artery (MCA). The brain is critically dependent on the continuous delivery of oxygen and glucose via the blood flow (7). Acute vascular occlusion interrupts the blood supply to the brain and can cause irreparable brain damage, resulting in cell death due to energy failure (8). As a rapid and non-invasive blood flow examination method (9, 10), transcranial Doppler (TCD) can monitor changes in the blood flow spectral waveforms, blood flow direction and velocity, and the pulsatility index (PI), allowing evaluation of the offending vessels and collateral branches (11, 12); TCD has been widely used in patients with ischemic stroke. Compared with diagnosis using cerebral angiography, the diagnosis of intracranial ICA and MCA mainstem occlusion using TCD showed a sensitivity and specificity of 95.0 and 92.0%, respectively (13). TCD plays an important role in the evaluation of patients with LHI due to its unique ability to evaluate hemodynamics in real time (14, 15).

In addition, in patients with a poor prognosis of LHI, the ischemic core region is significantly larger, cerebral blood perfusion is worse, and irreversible neuronal damage is more serious (16). Electroencephalograms (EEGs) can reflect changes in the metabolism and electrical activity of cortical neurons (17). When cerebral blood flow (CBF) becomes compromised, the EEG shows a loss of fast frequencies and an increase in slow frequencies. As CBF continues to decline toward the infarct threshold, irreversible damage occurs in neurons and the EEG becomes silent (18–20). Quantitative EEGs (QEEGs) transform the basic elements of EEGs into various quantitative parameters, being more objective and simpler than traditional EEGs for assessing brain damage in patients (21, 22). Most QEEG indicators [including relative alpha, theta, delta power, delta/alpha ratio (DAR), the (delta + theta)/(alpha + beta) ratio (DTABR), and brain symmetry index (BSI)] have been reported to be significantly associated with patient outcomes (23–27). These parameters showed a better prediction effect than the National Institutes of Health Stroke Scale (NIHSS) and ischemic volume (28, 29). The past two decades have seen rapid development in QEEG for the assessment of ischemic stroke outcomes and QEEG is considered as a powerful tool for prognostic prediction (30, 31).

The combination of TCD and QEEG as a new multimodality monitoring method is of major interest, as together, they describe stroke-related blood perfusion injury and neuronal function damage. In this study, we sought to explore whether it is possible to obtain more accurate and comprehensive prognostic predictors for patients with LHI by TCD-QEEG. Identifying such predictors is pivotal for developing risk-optimized treatment strategies and deciding on healthcare resource allocation.

## Materials and Methods

### Subjects

We conducted a prospective observational study between July 2018 and December 2019. Patients with LHI admitted to the Department of Neurology, First Hospital of Jilin University, China, were eligible for enrollment. The inclusion criteria were as follows: (1) admission time ≤ 72 h after LHI onset; (2) presence of LHI as confirmed by an experienced neurologist; (3) CT and/or MRI documenting infarction in at least two-thirds of the unilateral MCA territory and at least part of the basal ganglia, with or without additional ipsilateral infarction of the anterior or posterior cerebral artery (32); and (4) carotid ultrasound and TCD examination. The exclusion criteria were as follows: (1) patients who dropped out of the study; (2) inaccessible ultrasound windows; (3) intravascular thrombolysis or stent therapy; (4) significant contralateral infarction; (5) space-occupying hemorrhagic transformation of the infarct; (6) surgical decompression; and (7) intake of central nervous system depressants including sedatives, narcotics, antidepressants, antipsychotics, and antiepileptics. We also recruited 29 healthy age- and sex-matched controls (62 ± 10 years; 20 males), with normal brain MRI findings, no metabolic derangements, and normal central nervous system functioning.

This study was approved by the Ethics Committee of the First Hospital of Jilin University, China (NO. 2018-406) and conformed to the tenets of the Declaration of Helsinki. A written informed consent was obtained from the participants or next of kin of the participants before the commencement of this study.

### Clinical Data

The following variables of patients admitted to the neurological intensive care unit were recorded during monitoring: demographic characteristics, risk factors, vital signs, laboratory indices, the Glasgow Coma Scale (GCS) score, the NIHSS score, infarction side, and time from onset to monitoring. All the patients were treated according to the Guidelines for the Early Management of Patients with Acute Ischemic Stroke 2019 of the American Heart Association/American Stroke Association and received regular clinical care and postdischarge guidance (33).

Patients were followed-up 90 days after stroke onset by telephonic interview. The modified Rankin Scale score was used to determine the prognoses of the patients. The survival duration after disease onset was also recorded, if the patient had died.

### Non-Invasive Vascular Ultrasound Examination

The TCD criteria for the diagnosis of lesions at different locations were based on the criteria published by the European Society of Neurosonology and Cerebral Hemodynamics (34). When the site of severe stenosis or occlusion was located in the MCA, the primary TCD criterion was a blood flow spectrum in line with the thrombolysis in brain ischemia (TIBI) grades 0–4 at depths of < 45 mm (M2 segment) and 45–65 mm (M1 segment). For the detection of severe stenosis or occlusion in the terminal ICA (TICA), the primary TCD criteria included: (1) a blood flow spectrum in line with the TIBI grades 0–4 at a depth of 60–70 mm and (2) cross-filling of the anterior communicating artery (ACoA) or collateral flow in the posterior communicating artery (PCoA) indicated by increased velocity and carotid compression tests. When the site of severe stenosis or occlusion was located in the proximal ICA, the primary TCD criteria included: (1) cross-filling of the ACoA or collateral flow in the PCoA indicated by increased velocity and carotid compression tests; (2) reversed ophthalmic artery (OA) flow; and (3) delayed systolic flow acceleration, or blunted ipsilateral MCA, and mean flow velocity > 20 cm/s.

The TCD examination was performed using an EMS-9A (Delica, Shenzhen, China) device. To improve accuracy, the TCD-based diagnosis was referenced to the results of carotid ultrasound performed using the Philips CX50 ultrasound machine (Philips, Bothell, Washington, USA) (35).

### Transcranial Doppler-QEEG Measurements

Quantitative brain function monitoring using NSD version 1.0 (NSD-8100; Delica, Birsfelden, Switzerland) was performed with patients in the supine position and the head of the bed raised by 15–30°. TCD was performed using 2-Hz pulsed-wave probes fixed to each of the temporal windows of the patient via a helmet. A depth of 50–60 mm was used to acquire optimal MCA signals. Simultaneously, EEG was performed using Ag/silver chloride (AgCl) scalp electrodes positioned at 16 sites defined by the International 10–20 system, which uses 16 channels (Fp1, Fp2, F3, F4, F7, F8, C3, C4, T3, T4, T5, T6, P3, P4, O1, and O2) with the frontal central zero (Fz) electrode pole used for grounding and Cz, A1, and A2 used as references. The background EEG signal acquisition parameters were set as follows: time baseline, 30 mm/s; sensitivity, 10 μv/mm; electrode impedance maintained below 10 KΩ; sampling frequency, 250 Hz; and data filtering (high pass, 0.3 Hz; low pass, 30 Hz) (36). Each participant rested with their eyes closed until clear and stable data were achieved and data were then recorded for over 30 min. Every participant underwent monitoring only once.

### Transcranial Doppler-QEEG Original Data and Data Analysis

All the clearly readable TCD waveforms were used in the calculations. The following variables of flow velocity were analyzed from the TCD-QEEG machine: systolic flow velocity (Vs), diastolic flow velocity (Vd), mean velocity (Vm), and PI from the affected and unaffected hemispheres. The flow velocity of the MCA was classified into five stages according to TIBI grades 0–4 for further statistical analysis (37).

Electroencephalography artifact interpretation was performed independently by two physicians qualified in EEG interpretation. Artifact-free 5-min EEG segments were selected manually and quantitatively analyzed offline using MATLAB (MathWorks, Natick, Massachusetts, USA). The power spectral density was calculated using Fast Fourier transform for each electrode over the 1–30 Hz range. Power spectral density was calculated using Welch's averaged, modified periodogram spectral estimation method, with a 2-s Hamming window and 50% overlap. The frequency resolution was 0.5 Hz. From the resulting power spectra for each electrode, the absolute band power was summed across the delta (1–3 Hz), theta (4–7 Hz), alpha (8–13 Hz), and beta (14–30 Hz) bands (inclusive). Relative band power [relative delta power (RDP), relative theta power (RTP), relative alpha power (RAP), and relative beta power (RBP)] were calculated as the ratio of the summed absolute band power to the total summed power across the 1–30 Hz range (36). DAR and DTABR were calculated as the ratio of absolute power for the respective frequency bands of interest. The BSI was calculated as reported in previous studies (38, 39). Statistical differences in the above indicators among healthy controls, survivors, and non-survivors were analyzed.

### Statistical Analysis

A blinded analysis was performed for each patient. Data are reported as means and SDs of normally distributed variables and as medians and interquartile ranges (IQRs) for non-normally distributed variables. Categorical variables are presented as percentages. The Student's *t*-test was used for normally distributed variables. Non-parametric tests were used for non-normally distributed variables. Categorical variables were compared using the chi-squared test. After a preliminary analysis of prognoses of the patient, a survival analysis was performed using the duration of survival at 90 days after LHI onset. The parameters that had stronger correlations with the outcome under investigation were dichotomized for further analysis. Consequently, statistically significant (*p* < 0.05) parameters in the above analysis were entered into the Cox proportional-hazards model. The most economical model was obtained by backward stepwise elimination of the non-significant factors. The significance of variables in the multivariate models was evaluated using the Kaplan–Meier curves with log-rank tests. The Kaplan–Meier curves were plotted using the survminer package of RStudio (RStudio Incorporation, Boston, Massachusetts, USA) (https://CRAN.R-project.org/package=survminer). Moreover, receiver operating characteristic (ROC) curve analysis was used to assess the predictive ability of variables in the final model based on the area under the ROC curve (AUROC). The ROC curves were compared using DeLong's test.

All the statistical testing was two-tailed and a *p* < 0.05 was considered as statistically significant. Statistical analyses were performed using the SPSS version 22.0 (SPSS Incorporation, Chicago, Illinois, USA), RStudio version 1.2.1335 (RStudio Incorporation, Boston, Massachusetts, USA), and MedCalc version 19.0.7 (MedCalc Software, Mariakerke, Belgium, UK).

## Results

### Baseline Demographics

A total of 70 patients were originally recruited. Among them, 11 patients were excluded-−2 patients were lost to follow-up, 4 patients had disturbed EEG data, and 5 patients had poor penetration in the temporal window. The remaining 59 patients with LHI (63 ± 11.2 years; 40 males) were included in this study. Of these, 37 (67.3%) patients died by the 90-day follow-up. Characteristics of the patient are shown in Table 1. No significant differences were observed between survivors and non-survivors in terms of demographics or risk factors. With respect to the indicators of vital signs, heart rate (*p* = 0.010), respiratory rate (*p* = 0.013), and oxygen saturation (*p* = 0.021) differed significantly according to patient outcomes. The GCS (*p* = 0.001) and the NIHSS scores (*p* < 0.001) were significantly associated with mortality.

**Table 1:** Baseline characteristics.

**Characteristic**	**All patients (*n* = 59)**	**Non-survivors *n* = 22)**	**Survivors (*n* = 37)**	***P*-value**
**Demographics**				
Age (years), mean (SD)	63 (11.2)	67 (10.0)	61 (11.5)	0.056
Male sex, *n* (%)	40 (67.8)	12 (54.5)	28 (75.7)	0.093
**Risk factors**, ***n*** **(%)**				
Hypertension	30 (50.8)	9 (40.9)	21 (56.8)	0.239
Diabetes mellitus	11 (18.6)	5 (22.7)	6 (16.2)	0.731
Coronary heart disease	26 (44.1)	12 (54.5)	14 (37.8)	0.211
Smoking	36 (61.0)	13 (59.1)	23 (62.2)	0.815
Excessive drinking	30 (50.8)	9 (40.9)	21 (56.8)	0.239
Previous cerebral infarction	24 (40.7)	10 (45.5)	14 (37.8)	0.565
Previous cerebral hemorrhage	4 (6.8)	0 (0)	4 (10.8)	0.286
CVD family history	13 (22.0)	4 (18.2)	9 (24.3)	0.749
**Vital signs**				
Temperature, median (IQR)	37.0 (0.55)	37.0 (0.80)	36.9 (0.50)	0.275
Heart rate, mean (SD)	86 (19.6)	94 (22.4)	81 (16.0)	0.010
SBP (mm Hg), mean (SD)	149 (20.5)	152 (21.1)	148 (20.4)	0.479
DBP (mm Hg), mean (SD)	85 (16.7)	85 (19.2)	84 (15.2)	0.795
Respiratory rate, median (IQR)	20 (8.5)	23.0 (8.0)	18.5 (6.0)	0.013
Oxygen saturation, median (IQR)	100 (1)	99 (2)	100 (1)	0.021
**Laboratory indexes**				
WBC (× 10^9^/L), mean (SD)	11.63 (3.55)	11.98 (3.33)	11.42 (3.70)	0.567
PLT (× 10^9^/L), mean (SD)	220 (62.6)	211 (60.1)	224 (66.4)	0.464
APTT (s), mean (SD)	29.1 (4.66)	30.1 (5.34)	28.6 (4.29)	0.307
INR, mean (SD)	1.03 (0.11)	1.05 (0.99)	1.02 (0.12)	0.366
Glucose (mmol/L), median (IQR)	6.72 (2.36)	7.78 (3.35)	7.09 (2.58)	0.638
HbA1c, median (IQR)	5.7 (1.2)	5.6 (2.6)	5.8 (0.9)	0.910
HCY (μmol/L), median (IQR)	17.4 (11.3)	16.7 (8.6)	17.6 (16.0)	0.389
Vitamin B12 (mg/L), median (IQR)	207 (214.5)	257 (208.0)	183 (211.0)	0.077
**GCS score** **≤** **8**, ***n*** **(%)**	19 (32.2)	13 (59.1)	6 (16.2)	0.001
**NIHSS score** **>** **25**, ***n*** **(%)**	13 (22.0)	11 (50.0)	2 (5.4)	<0.001
**Infarction side, left**, ***n*** **(%)**	35 (59.3)	15 (68.2)	20 (54.1)	0.285
**Time from onset to monitoring (h), mean (SD)**	52.07 (38.64)	44.73 (34.18)	56.43 (40.88)	0.264

*CVD, cerebrovascular disease; IQR, interquartile range; SBP, systolic pressure; DBP, diastolic pressure; WBC, white blood cell; PLT, platelet; APTT, activated partial thromboplastin time; INR, international normalized ratio; HbAlc, glycosylated hemoglobin; HCY, homocysteine; GCS, Glasgow Coma Scale; NIHSS, National Institutes of Health Stroke Scale*.

### Non-Invasive Vascular Ultrasound Examination

As shown in Table 2, prognosis correlated significantly with the offending vessel (*p* = 0.005). Among the patients who had died, the offending vessel was most often the TICA [7 of 8 (87.5%) patients], followed by the MCA [8 of 22 (36.4%) patients], and the proximal ICA [7 of 29 (24.1%) patients]. Thus, the TICA as the offending vessel was strongly associated with the mortality rate.

**Table 2:** Findings of the conventional ultrasound examination.

**Offending vessel, *n* (%)**	**Non-survivors (*n* = 22)**	**Survivors (*n* = 37)**	**Total (*n* = 59)**	***P*-value**
Proximal ICA	7 (24.1)	22 (75.9)	29	0.005
TICA	7 (87.5)	1 (12.5)	8	
MCA	8 (36.4)	14 (63.6)	22	
**Collateral branch**, ***n*** **(%)**	**Non-survivors** **(*****n*** **=** **14)**	**Survivors** **(*****n*** **=** **23)**	**Total** **(*****n*** **=** **37)**	***P*** **-value**
Any branch exists	7 (30.4)	16 (69.6)	23	0.234
ACoA/PCoA presence	6 (37.5)	10 (62.5)	16	0.970
OA presence	1 (9.1)	10 (90.9)	11	0.027

*ICA, internal cerebral artery; TICA, terminal ICA; MCA, middle cerebral artery; ACoA, anterior communicating artery; PCoA, posterior communicating artery; OA, ophthalmic artery*.

Table 2 shows the results of statistical analysis for the involvement of collateral vessels in ICA stenosis/occlusion. There were no significant differences between groups according to the presence or absence of collateral circulation, the presence or absence of the ACoA, or the PCoA compensated through the circle of Willis. Importantly, of the 23 patients who survived 90 days, 10 patients had a collateral branch of the OA. In contrast, only 1 patient among the 14 patients who died within 90 days had a collateral branch of the OA. The presence of the collateral branch of the OA was significantly associated with mortality (*p* = 0.027).

### Original TCD-QEEG Data

**Figure 1:**
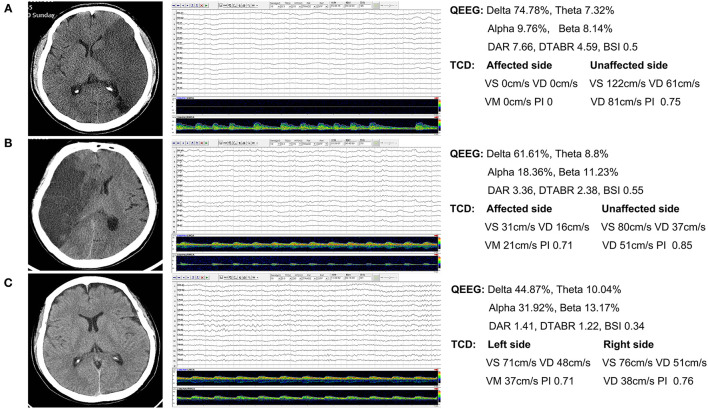
Representative patients. **(A)** Nonsurvivor: QEEG image shows that the slower delta frequency band is significantly increased and the faster alpha frequency band is significantly decreased. The DAR and DTABR are increased. The TCD image shows that the flow velocity of the affected side is notably decreased. **(B)** Survivor: QEEG and TCD images show similar changes. **(C)** Healthy control: QEEG and TCD images show normal findings. QEEG, quantitative electroencephalography; DAR, delta/alpha ratio; DTABR, (delta + theta)/(alpha + beta) ratio; BSI, brain symmetry index; TCD, transcranial Doppler; Vs, systolic flow velocity; Vd, diastolic flow velocity; Vm, mean velocity; PI, pulsatility index.

Figure 1 shows the CT and TCD-QEEG images of representative patients.

**Figure 2:**
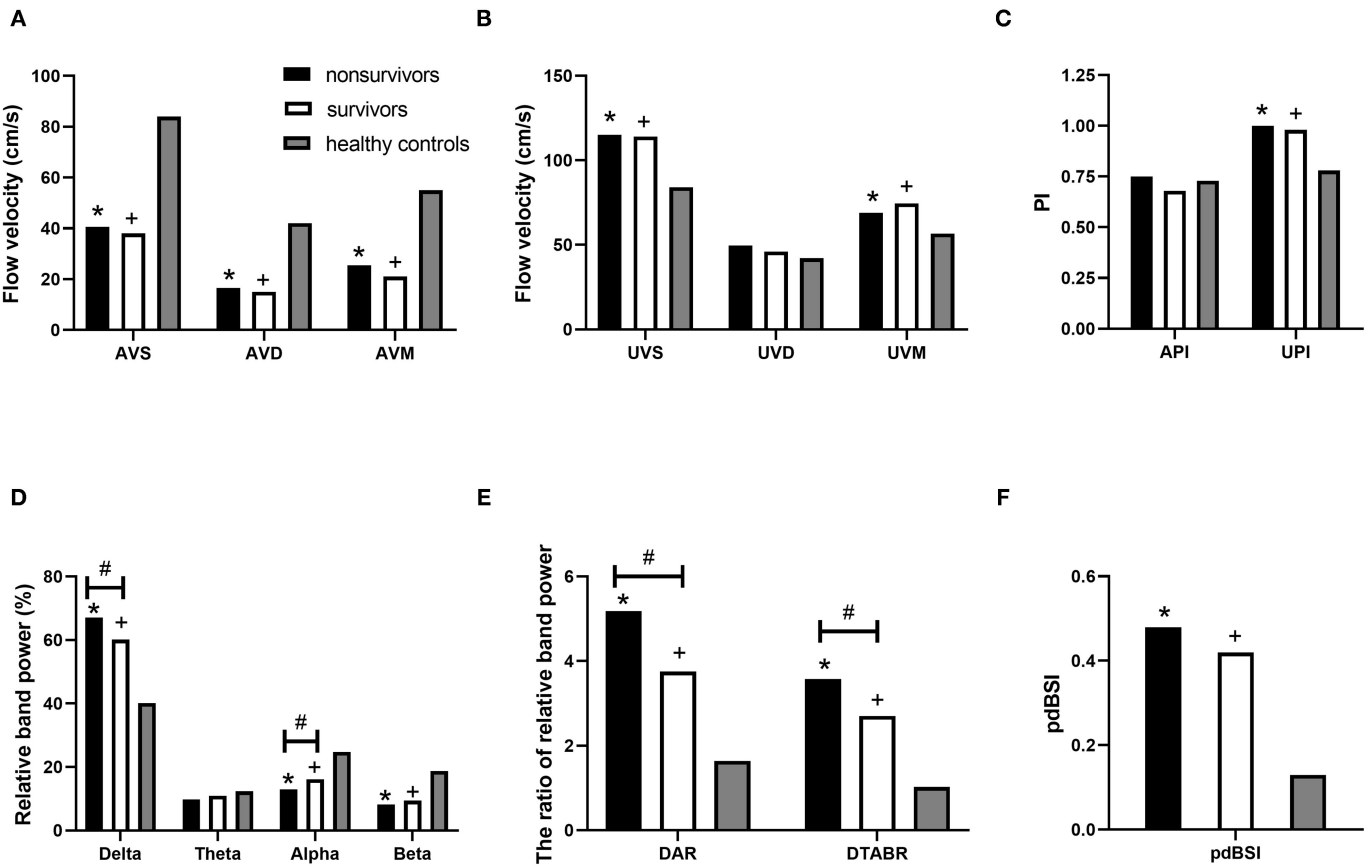
TCD-QEEG parameters for nonsurvivors, survivors, and healthy controls. TCD parameters: **(A)** AVS, AVM, and AVD; **(B)** UVS, UVM, and UVD; and **(C)** API and UPI. QEEG parameters: **(D)** relative delta power, relative theta power, relative alpha power, and relative beta power; **(E)** DAR and DTABR; and **(F)** BSI. *p < 0.05 for nonsurvivors vs. healthy controls; ^+^*p* < 0.05 for survivors vs. healthy controls; ^#^*p* < 0.05 for nonsurvivors vs. survivors. API, affected pulsatility index; AVD, affected diastolic flow velocity; AVM, affected mean flow velocity; AVS, affected hemisphere systolic flow velocity; DAR, delta/alpha ratio; DTABR, (delta + theta)/(alpha + beta) ratio; BSI, brain symmetry index; QEEG, quantitative electroencephalography; TCD, transcranial Doppler; UPI, unaffected pulsatility index; UVD, unaffected diastolic flow velocity; UVM, unaffected mean flow velocity; UVS, unaffected hemisphere systolic flow velocity.

Figure 2 and Table 3 show the Vs, Vd, Vm, and PI of the MCA on TCD. On the affected side, the Vs, Vd, and Vm were lower in patients than in the healthy controls (*p* < 0.05). On the unaffected side, the Vs, Vm, and PI were higher in the patients (*p* < 0.05). Non-survivors and survivors did not show significant differences in the aforementioned indicators. Blood flow velocity in the MCA, which was further classified into five stages according to the TIBI grades (0–4), did not significantly affect the clinical outcomes of the patients.

**Table 3:** TCD and QEEG parameters.

	**Non-survivors (*n* = 22)**	**Survivors (*n* = 37)**	**Healthy controls (*n* = 29)**
**TCD parameters**			
VS (cm/s)			
Affected side, median (IQR)	40.5 (78)^*^	38.0 (64)^+^	84.0 (19)
Unaffected side, median (IQR)	115 (48)^*^	114 (45)^+^	
VD (cm/s)			
Affected side, median (IQR)	16.5 (31)^*^	15.0 (29)^+^	42.0 (12)
Unaffected side, median (IQR)	49.5 (24)	46.0 (21)	
VM (cm/s)			
Affected side, median (IQR)	25.5 (48)^*^	21.0 (40)^+^	55.0 (16)
Unaffected side, mean (SD)	68.9 (18.4)^*^	74.6 (22.5)^+^	56.7 (12.9)
PI			
Affected side, median (IQR)	0.75 (1.04)	0.68 (0.87)	0.73 (0.20)
Unaffected side, mean (SD)	1.00 (0.17)^*^	0.98 (0.22)^+^	0.78 (0.17)
**QEEG parameters**			
RDP (%), mean (SD)	67.06 (7.53)^*#^	60.16 (8.93)^+^	40.19 (12.14)
RTP (%), median (IQR)	9.82 (5.39)	10.99 (4.50)	12.35 (7.63)
RAP (%), median (IQR)	12.88 (4.92)^*#^	16.15 (7.18)^+^	24.76 (10.62)
RBP (%), median (IQR)	8.19 (3.11)^*^	9.44 (5.42)^+^	18.76 (11.32)
DAR, median (IQR)	5.18 (2.67)^*#^	3.75 (1.99)^+^	1.64 (1.46)
DTABR, median (IQR)	3.57 (1.34)^*#^	2.70 (1.54)^+^	1.02 (1.05)
BSI, median (IQR)	0.48 (0.09)^*^	0.42 (0.14)^+^	0.13 (0.25)

*TCD, transcranial Doppler; QEEG, quantitative electroencephalography; VS, systolic flow velocity; IQR, interquartile range; VM, mean flow velocity; VD, diastolic flow velocity; PI, pulsatility index; RDP, relative delta power; RTP, relative theta power; RAP, relative alpha power; RBP, relative beta power; DAR, delta/alpha; DTABR, (delta + theta)/(alpha + beta); BSI, brain symmetry index*.

* *p < 0.05 for non-survivors vs. healthy controls;*

+ *p < 0.05 for survivors vs. healthy controls; and*

# *p < 0.05 for non-survivors vs. survivors*.

Figure 2 and Table 3 list the QEEG indicators. RDP, DAR, DTABR, and BSI were higher, while RAP and RBP were lower in patients than in healthy controls (*p* < 0.05). In both the non-survivors and survivors, higher RDP, DAR, and DTABR and lower RAP were associated with mortality (*p* < 0.05).

### Survival Analysis

**Figure 3:**
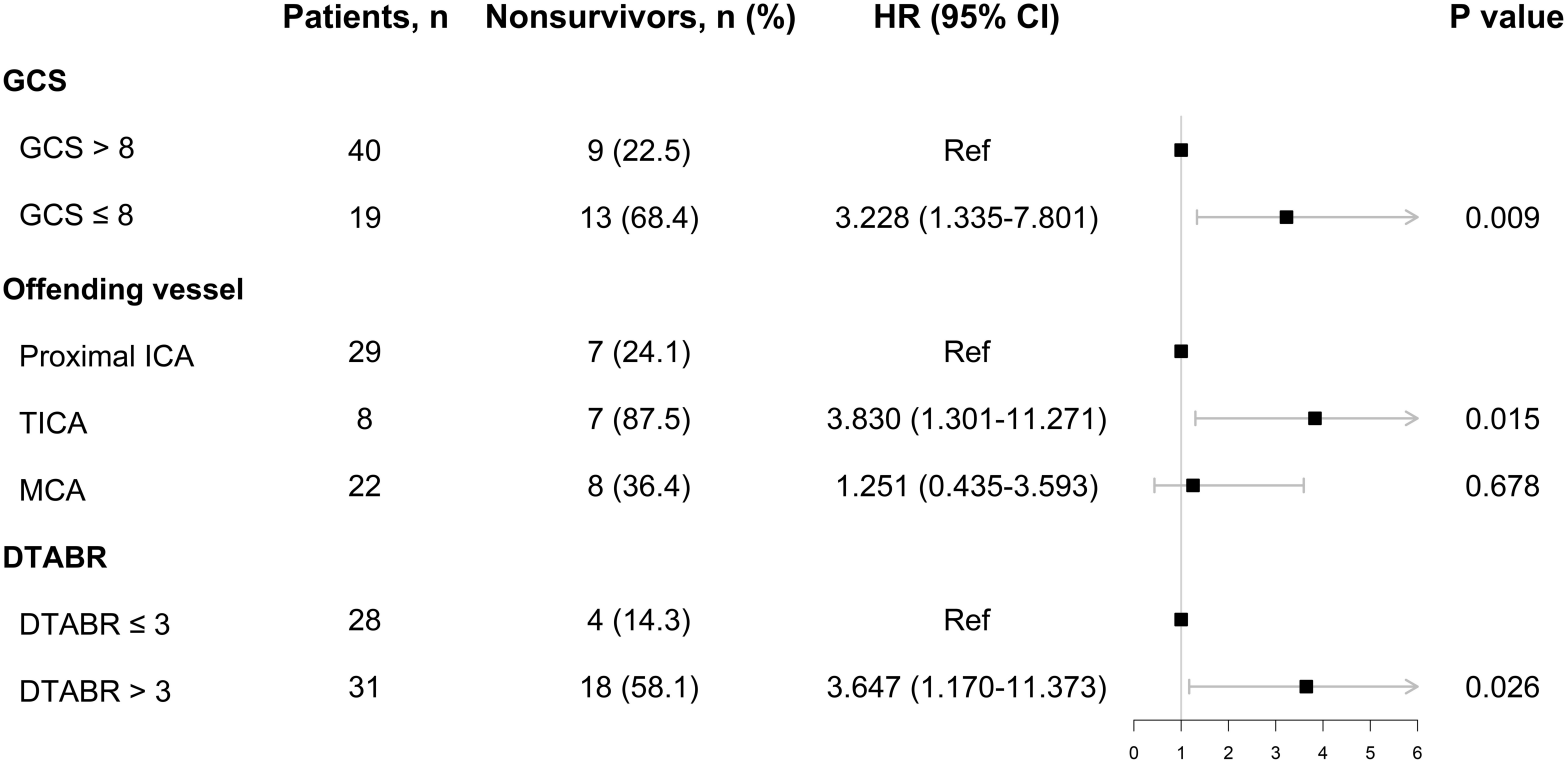
The Cox proportional-hazards model. DTABR, (delta + theta)/(alpha + beta) ratio; GCS, Glasgow Coma Scale; HR, hazard ratio; ICA, internal carotid artery; MCA, middle cerebral artery; TICA, terminal ICA.

The Cox proportional-hazards model incorporated all the variables with *p* < 0.05 from the above analyses. With respect to QEEG variables, DAR and DTABR were selected for further analysis: patients were divided into two groups based on a threshold DAR of 4.0 or a threshold DTABR of 3.0. The final Cox proportional-hazards model incorporated the GCS score, the offending vessel, and DTABR. As shown in Figure 3, the GCS score of ≤ 8 [hazard ratio (HR), 3.228; 95% CI, 1.335–7.801; *p* = 0.009], the TICA as the offending vessel (HR, 3.830; 95% CI, 1.301–11.271; *p* = 0.015), and DTABR ≥ 3 (HR, 3.647; 95% CI, 1.170–11.373; *p* = 0.026) are the three independent indicators of survival duration identified.

**Figure 4:**
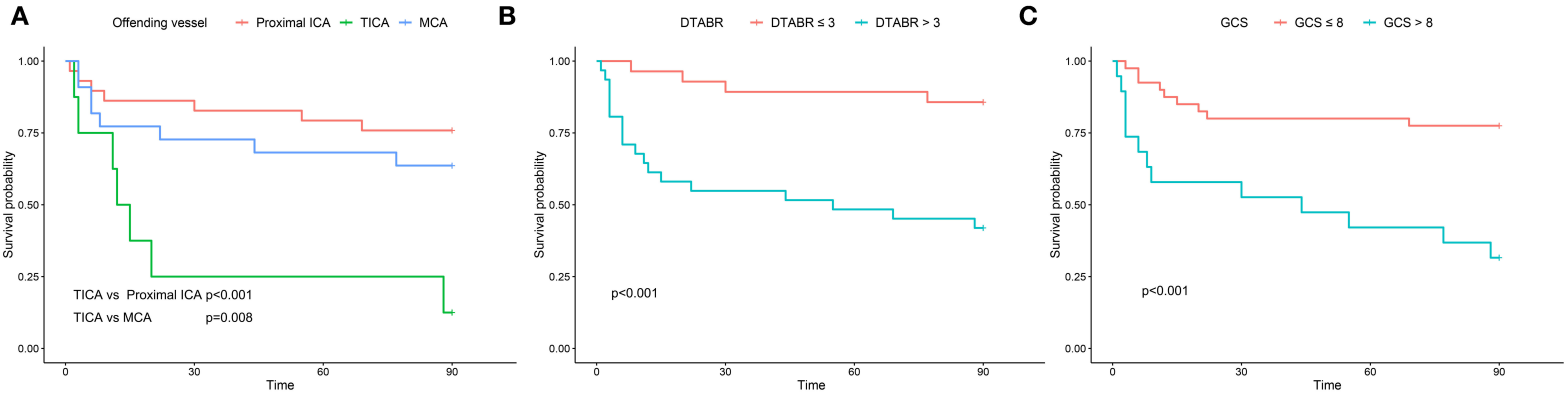
The Kaplan–Meier survival curves for the training cohort. **(A)** The Kaplan-Meier survival curves for the offending vessel. **(B)** The Kaplan-Meier survival curves for DTABR. **(C)** The Kaplan-Meier survival curves for GCS. ICA, internal carotid artery; TICA, terminal internal carotid artery; MCA, middle cerebral artery; DTABR, (delta + theta)/(alpha + beta) ratio; GCS, Glasgow Coma Scale.

The Kaplan–Meier curves were plotted for the GCS score, the offending vessels, and DTABR (Figure 4; log-rank *p* < 0.001, *p* = 0.001, and *p* < 0.001, respectively). The median survival time in those with the GCS ≤ 8 was 44 days, with TICA as the offending vessel was 12 days, and with a DTABR > 3 was 55 days.

### Receiver Operating Characteristic Curves

**Figure 5:**
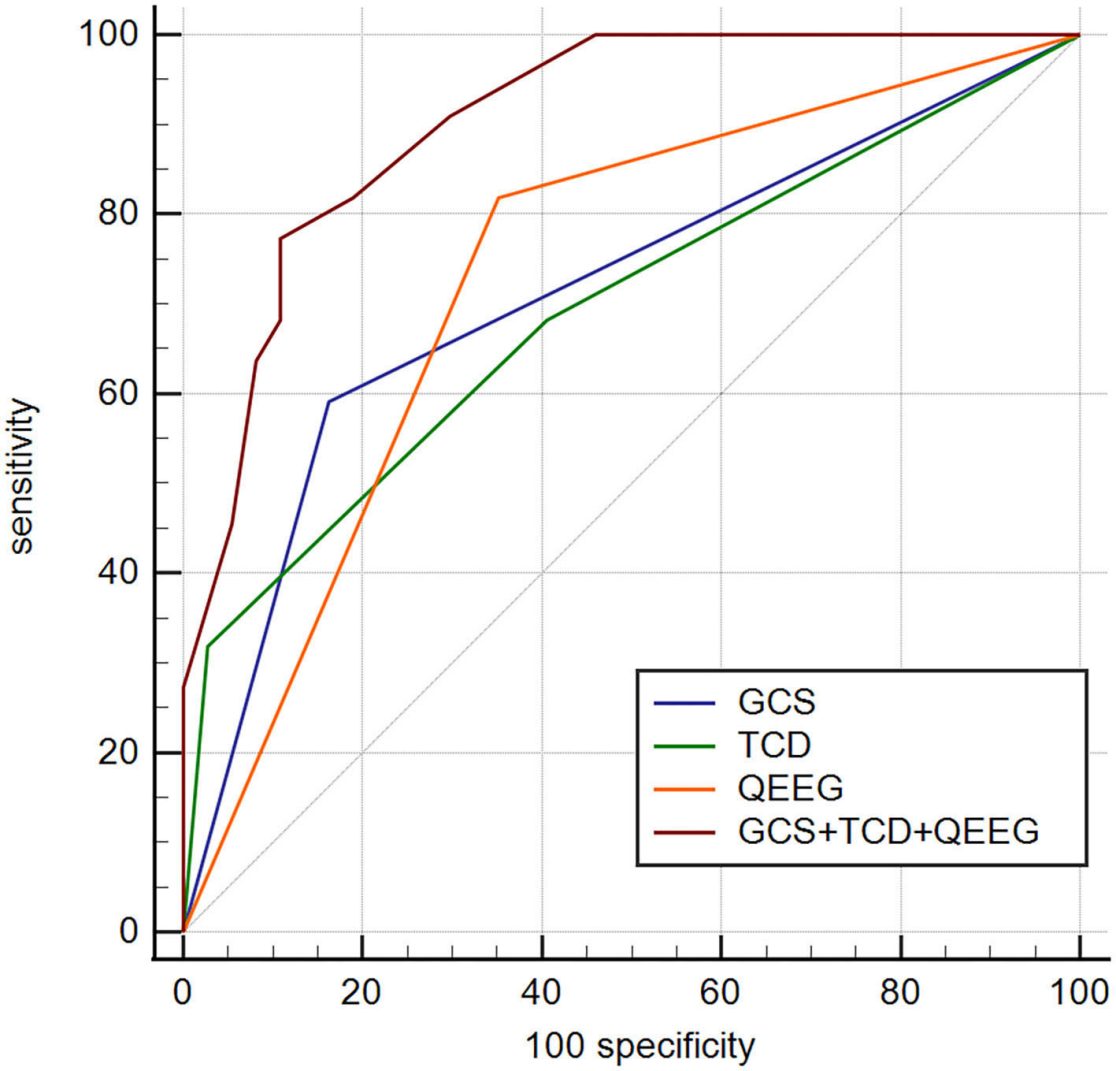
Comparison of ROC curves to predict outcome in this cohort between four models. GCS, AUROC 0.715 (0.582–0.824); TCD—offending vessel, AUROC 0.693 (0.560–0.807); QEEG—DTABR, AUROC 0.733 (0.602–0.840); GCS + TCD + QEEG, AUROC 0.905 (0.801–0.966). *p* < 0.05 for GCS + TCD (offending vessel) + QEEG (DTABR) comparison with GCS, offending vessel (independent predictor of TCD), and DTABR (independent predictor of QEEG). ROC, receiver operating characteristic; GCS, Glasgow Coma Scale; AUROC, area under the ROC curve; TCD, transcranial Doppler, QEEG, quantitative electroencephalography; DTABR, (delta + theta)/(alpha + beta) ratio; VD, diastolic flow velocity.

To determine whether TCD-QEEG variables combined with the GCS score in the model improved outcome prediction, we compared the ROC curves for four models. The first model was obtained using the GCS score, the second model was obtained using the TCD (the offending vessel), the third model was obtained by using the QEEG (DTABR), and the final model included the combination of GCS, TCD, and QEEG. The AUROC for the model that included GCS, TCD, and QEEG was 0.905; the sensitivity of this combination for predicting mortality within 90 days was 77.27% and specificity was 89.19%. Comparison of the ROC curves showed that the efficacy of the combination of GCS, TCD, and QEEG for predicting mortality in patients with LHI was better than that of the GCS score (AUROC 0.714; *p* = 0.0003), TCD (AUROC 0.693; *p* = 0.0009), or QEEG (AUROC 0.733; *p* = 0.0040). Therefore, the contribution of the final model was significant (Figure 5).

## Discussion

This study used a combination of TCD and QEEG to assess the prognosis of patients with LHI by evaluating changes in brain function; such an approach has not been used previously. We used TCD-QEEG as a new bedside monitoring approach and confirmed that the GCS score, TCD, and QEEG were independent predictors of 90-day mortality. Moreover, we showed that the combination of the GCS score, DTABR from the QEEG, and the offending vessel from TCD together showed a marked potential for predicting the prognosis in patients with LHI. Our findings emphasize the importance of monitoring the damage of CBF and neuronal activity when evaluating the prognosis of patients with LHI.

The GCS score is the most widely used indicator of the severity of consciousness disturbance (40). Many studies have shown that the GCS score was associated with a poor prognosis (41, 42). Patients with LHI usually demonstrate a severe disturbance of consciousness (43) and for patients with LHI, the baseline GCS score at admission has been significantly associated with death (44). We found that the GCS score of ≤ 8 significantly affected the duration of survival in patients with LHI. However, the subjectivity of the surgeons and dysphasia, mechanical ventilation, or sedation of patients may bias the results (45–47). Therefore, for a more accurate and comprehensive evaluation of patients, we suggest that the GCS score should be combined with the other variables included in our multivariate model to predict prognosis, as other studies have suggested previously (48, 49).

Quantitative electroencephalography, as a non-invasive and economical bedside monitoring method, can reflect the decrease of CBF and the change of neuron damage (50). The significant QEEG characteristics of patients with LHI are increased delta activity, decreased beta and alpha activity, and a decrease in the overall background activity (51). Additionally, Sheorajpanday et al. found that BSI is an independent predictor of acute anterior circulation syndrome (28). In this study, we found that the RDP, RAP, RBP, DAR, DTABR, and BSI of patients with LHI are significantly different from those of healthy controls. When comparing EEG findings with clinical outcomes, depression of alpha frequency together with continuous delta activity was a predictor of poor outcome (52). DAR and DTABR have important predictive value in the evaluation of patients with LHI (26, 27, 53). Compared with DAR and DTABR as a marker of global brain function, it can also be used to evaluate theta and beta activities. It can independently predict mortality in patients with LHI (28, 29). The data in this study support previous findings that RAP, RDP, DAR, and DTABR are all related to the mortality of patients with LHI within 90 days. In order to promote clinical translation and utility of the study findings, it is necessary to formulate standard ranges or standard values of variables. Studies have shown that the DAR threshold of 3.7 can distinguish ischemic stroke from healthy brains (53). In a study of 24 patients with lacunar cerebral infarction, DTABR < 1 had 100% specificity for the absence, while DTABR > 3.5 had 100% sensitivity for the presence of such lesions (54). This study found that DTABR > 3 was an independent predictor of survival duration in patients with LHI and could reliably distinguish patients with a poor prognosis. The application of QEEG is, thus, an indispensable part of multimodal monitoring in the clinical management of patients with severe LHI.

Patients with LHI primarily have three offending vessels: the proximal ICA, TICA, and MCA. In this study, TCD detected that severe stenosis or occlusion of the TICA was an independent predictor of the duration of survival in patients with LHI. The mortality rate in patients with TICA as the offending vessel was about 3.8 times that of patients with the proximal ICA as the offending vessel. Other researchers have also found that patients with ≥ 50% stenosis or occlusion, as a result of TICA atherosclerosis, had a higher risk of stroke and death and their long-term prognosis was poor compared to that of patients with proximal ICA involvement (55, 56). Moreover, patients with a hyperdense TICA sign caused by a thrombus also had a more severe initial neurological deficit and a worse prognosis (57). We reasoned that differences in prognosis between patients with TICA and proximal ICA might be related to compensation by the OA. A previous study has demonstrated that when the ICA is occluded, cerebral perfusion strongly depends on the contribution of the ipsilateral external carotid artery (58). This indicates that, in future clinical work, clinicians should pay more attention to the occurrence of severe TICA stenosis or occlusion, since TICA lesions are associated with a poor prognosis.

Pathophysiological changes in patients with LHI remain problematic. BSI was found to be an indicator of bilateral hemispheric damage symmetry (38, 59). In this study, BSI was only statistically significantly different between patients with LHI and healthy controls, but there was no difference between survivors and non-survivors. In the study of Bentes et al. (29), DTABR and RAP, rather than BSI, were the best QEEG indicators of prognosis in patients with LHI. Therefore, the predictive effect of BSI requires further studies in patients with LHI. Monitoring of dynamic changes in variables may provide more insight. In addition, TCD monitoring in the first 6 or 12 h after admission has shown that lack of blood flow or asymmetric flow velocity in the MCA is an independent predictor of poor prognosis (60, 61). This study found no predictive significance, perhaps due to later monitoring. It is possible that cerebrovascular recanalization or early reocclusion had occurred; therefore, in this study, the offending vessels, rather than flow velocity, were the ultimate predictor.

The results of this study should be interpreted with caution, given its limitations. First, this study had a small sample size. Second, although participants with LHI were included, specific differences in the volume of the cerebral infarction may also have influenced the outcome. Third, this study found only a predictive effect of TCD-QEEG on mortality of the patient; the predictive effect of TCD-QEEG on the quality of life of the patient warrants further investigation. Finally, the brain function of patients should be dynamically monitored at an earlier point in future studies to determine if changes therein are associated with outcomes.

Our results show that the GCS score, QEEG, and TCD can independently predict poor prognosis in patients with LHI, which has not been reported previously. Combining the clinical assessment scale and multimodal monitoring methods yielded the best prognostic prediction model that could provide more accurate prognostic information on mortality risk. Based on the advantages of non-invasive and bedside monitoring, this is expected to become a routine method to evaluate the prognosis of patients with LHI.

## Data Availability Statement

The raw data supporting the conclusions of this article will be made available by the authors, without undue reservation.

## Ethics Statement

The studies involving human participants were reviewed and approved by the Ethics Committee of the First Hospital of Jilin University, China (NO. 2018-406). The patients/participants provided their written informed consent to participate in this study.

## Author Contributions

YQ and LW contributed to the conceptualization, methodology, formal analysis and investigation, and writing–original draft preparation. YX contributed to the conceptualization, methodology, formal analysis and investigation, writing–review and editing, funding acquisition, and resources. JZ, YCa, and LL contributed to the methodology, formal analysis, and investigation. YCh contributed to the conceptualization, writing–review and editing, funding acquisition, resources, and supervision. All the authors read and approved the final manuscript.

## Funding

This study was supported by the National Natural Science Foundation of China (Grant No. 82102066 and No. 81971620) and the Natural Science Foundation of Jilin Science and Technology Department (Grant No. 20210101254JC and 20200201522JC).

## Conflict of Interest

The authors declare that the research was conducted in the absence of any commercial or financial relationships that could be construed as a potential conflict of interest.

## Publisher's Note

All claims expressed in this article are solely those of the authors and do not necessarily represent those of their affiliated organizations, or those of the publisher, the editors and the reviewers. Any product that may be evaluated in this article, or claim that may be made by its manufacturer, is not guaranteed or endorsed by the publisher.
